# Genotypic variation in intrinsic transpiration efficiency correlates with sugarcane yield under rainfed and irrigated field conditions

**DOI:** 10.1111/ppl.13221

**Published:** 2020-10-28

**Authors:** Sijesh Natarajan, Jaya Basnayake, Prakash Lakshmanan, Shu Fukai

**Affiliations:** ^1^ School of Agriculture and Food Sciences University of Queensland St Lucia Queensland Australia; ^2^ Sugar Research Australia Brandon Queensland Australia; ^3^ Sugarcane Research Institute Guangxi Academy of Agricultural Sciences Nanning China; ^4^ Interdisciplinary Research Center for Agriculture Green Development in Yangtze River Basin (CAGD) College of Resources and Environment, Southwest University Chongqing China; ^5^ Queensland Alliance for Agriculture and Food Innovation University of Queensland St Lucia Queensland Australia

## Abstract

Intrinsic transpiration efficiency (_i_ *TE*), the ratio of photosynthesis (*A*) to stomatal conductance (*g*_s_), is considered a useful trait for improving productivity; however, higher _i_ *TE* with high *A* is more desirable than that with low *g*_s_. This study dissects _i_ *TE* of 20 sugarcane genotypes to understand its relationship with total dry matter (TDM) and cane yield (TCH) under irrigated and rainfed conditions. Water stress reduced mean *A* and *g*_s_ by 56 and 61%, and mean TDM and TCH by 55 and 59%, respectively; however, genotype × irrigation treatment interaction was smaller than genotype variance. Mean _i_ *TE* increased from 117.4 μmol mol^−1^ in the irrigated treatment to 130.6 μmol mol^−1^ in the rainfed treatment. In irrigated conditions, _i_ *TE* had high heritability (*H* ^2^_b_ = 0.67) and significant genetic correlation with TDM (*r*_g_ = 0.58) and TCH (*r*_g_ = 0.72). Under water stress, at *g*_s_ below 0.1 mol m^−2^ s^−1^, non‐stomatal limitation to *A* was evident and _i_ *TE* had low heritability (*H* ^2^_b_ = 0.2). Whereas in the *g*_s_ range of 0.1–0.4 mol m^−2^ s^−1^, heritability of _i_ *TE* (*H* ^2^_b_ = 0.63) and its genetic correlation with TDM (*r*_g_ = 0.78) and TCH (*r*_g_ = 0.75) were maximised. There was significant genotypic variation in photosynthetic capacity (*A*_c_), and the differences were related to TDM and _i_ *TE*. Selecting genotypes with higher _i_ *TE* and *A*_c_ could offer potential for improving productivity without the unfavourable effect of low *g*_s_.

**Abbreviations**
*A*: photosynthesis
*A*_c_: photosynthetic capacity
*C*_i_: intercellular CO_2_ concentration
*g*_s_: stomatal conductance
IR: irrigated treatment
_i_*TE*: intrinsic transpiration efficiency
RF: rainfed treatment
TCH: tonnes cane yield per hectare
TDM: total dry matter
*TE*: transpiration efficiency
WUE: water use efficiency

## 1 INTRODUCTION

Sugarcane is a major crop grown in over 110 countries to meet most of the world's demand for sugar as well as a substantial portion of the demand for bioenergy. The global prevalence of sugarcane has been attributed to its high productivity and adaptability to both tropics and sub‐tropics. However, sugarcane also has high water demand for profitable production with an average evapotranspiration rate of 7–8 mm day^−1^ when grown with adequate water and nutrition (Inman‐Bamber and Smith, 2005). Water stress is one of the most important limitations to sugarcane productivity worldwide (Inman‐Bamber and Smith, 2005). In Australia, the average loss in sugarcane production due to water stress is around 15% per annum, an estimated A $230 million economic loss (Inman‐Bamber, 2007). Restrictions on water for irrigation or rising costs of irrigation, erratic rainfall, and recurring and increasingly prolonged drought due to climate change (Park and Attard, 2005) is further constraining sugarcane productivity. Therefore, improving water use efficiency (WUE), the ratio of dry matter production to water use, is becoming increasingly important in sugarcane production. While management practices have been employed over the years to improve WUE, there may be potential for genetic improvement of WUE in sugarcane without compromising productivity (Inman‐Bamber *et al*., 2012; Jackson *et al*., 2016).

WUE can be defined at different levels. At the field‐level, WUE is defined as the ratio of dry matter accumulation to water supply via rainfall and irrigation. At the plant‐level, WUE is often reported as transpiration efficiency (*TE*) and is defined as dry matter accumulation per unit water transpired. At the leaf‐level, WUE is defined as intrinsic transpiration efficiency (_i_ *TE*), the ratio of photosynthesis (*A*) to stomatal conductance (*g*_s_) (Condon *et al*., 2002). Passioura (1996) defined yield using a simple framework as the product of water use and WUE, suggesting any increase in WUE without decreasing water use will improve productivity. However, incorporating WUE as a selection target in breeding programmes has had little success apart from the water use efficient wheat varieties ‘Drysdale’ and ‘Rees’ (Rebetzke *et al*., 2002). Challenges arise due to a possible negative correlation between WUE and yield when higher WUE results from reduced water use, especially when grown in well‐watered conditions (Blum, 2009). The lack of a simple, cost‐effective technique for measurement WUE in the field also hinders its application in breeding programmes. Although the leaf‐level _i_ *TE* can be measured reliably using instantaneous infra‐red gas exchange, it is a physiologically complex trait resulting from the combined expression of multiple physiological processes and parameters, such as *A*, *g*_s_ and leaf intercellular CO_2_ concentration (*C*_i_), and many other processes that determine carbon balance and growth, which are easily influenced by the growing environment (Sinclair, 2012). A better understanding of the component physiological mechanisms that determine genotypic variation in _i_ *TE* and its interaction with typical growing environment may render the trait amenable to crop improvement.

Significant genetic variation in sugarcane yield under water stress has been reported in numerous studies (Smit and Singels, 2006; Basnayake *et al*., 2012; De A. Silva *et al*., 2012; Hemaprabha *et al*., 2012); however, the physiological mechanism underpinning the genetic variation is not well understood. In a study involving multi‐environment trials in rainfed and irrigated conditions, *g*_s_ showed a range of genetic correlation from −0.29 to 0.94 with cane yield depending on the environmental conditions (Basnayake *et al*., 2015). The significant genetic correlations in certain environments indicate that *g*_s_ is at least partly a significant determinant of genotype yield. However, the physiological basis of the poor genetic correlations observed in other environments is not understood. In a theoretical assessment, targeted selection of sugarcane varieties with higher _i_ *TE* was proposed as a way of increasing productivity in rainfed regions and improving yield stability in regions with erratic rainfall (Inman‐Bamber *et al*., 2012). Another modelling study showed that for every percentage increase in *TE*, cane yield increases by an average 0.5–0.9% in the Australian rainfed environments (Stokes *et al*., 2016). A pot‐based study with genetically diverse sugarcane genotypes showed significant genotypic variation in *C*_i_ (Jackson *et al*., 2016). However, despite the increasing knowledge of the potential value of _i_ *TE*, genotypic variation in _i_ *TE* and its influence on sugarcane growth and productivity under commercially‐relevant field conditions have not been studied previously.

Increase in _i_ *TE* can arise either due to higher *A* or lower *g*_s_ (Condon *et al*., 2004); however, higher _i_ *TE* with lower *g*_s_ may result in reduced water use, which could be beneficial in severely water stress conditions but can have yield penalties in well‐watered environments due to their inability to use all available soil moisture (Sinclair, 2012). Whereas higher _i_ *TE* resulting from higher *A* will be beneficial for most environments, particularly in intermittent moderate water stress scenarios commonly experienced in most sugarcane production conditions in Australia (Ghannoum, 2016; Jackson *et al*., 2016). Variation in _i_ *TE* can be influenced by the position of genotype on the *A* versus *g*_s_ curvilinear relationship curve, determined by the growing environment, and the inherent capacity of the genotype to assimilate CO_2_ at a given *g*_s_ (i.e., genotypic effect in a given environment). Gilbert *et al*. (2011) suggested one way to overcome the trade‐off between high _i_ *TE* and low *g*_s_ is to select for _i_ *TE* which arises due to higher photosynthetic capacity (*A*_c_) at a given *g*_s_. In sugarcane, little is known about the variation in components of _i_ *TE* and how it interacts with the intensity and duration of water stress in natural growing conditions to determine _i_ *TE*.

Understanding the extent of genotypic variation in _i_ *TE* and its relationship with productivity, and the underpinning physiological mechanisms under commercially‐relevant irrigation regimes is critical to assess the prospect of using _i_ *TE* as a selection target in sugarcane crop improvement. The objectives of this study were, (1) to investigate genotypic variation for total dry matter (TDM), tonnes cane yield per hectare (TCH) and _i_ *TE*, (2) to determine the relationship between _i_ *TE* and crop productivity under rainfed and irrigated conditions, and (3) to determine if variation in *A*_c_ exists independent of *g*_s_, which can be exploited to improve _i_ *TE* without the negative effects of reduced *g*_s_.

## 2 MATERIALS AND METHODS

### 2.1 Experimental conditions

**FIGURE 1:**
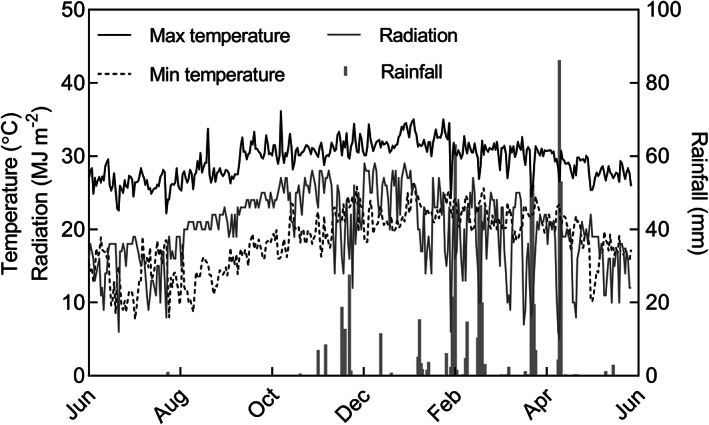
Weather conditions at the experiment site between June 2013 and June 2014

A field experiment was conducted in Brandon, Queensland (19° 56′S 147° 36′E), Australia from June 2013 to June 2016. Cane yield data from the 3 years in this experiment was reported previously, which indicated genotype × crop‐year interaction variance was small compared to genotype variance (Natarajan *et al*., 2020). In this study, we report cane yield and associated agronomic and physiological attributes from the first year (2013–2014). The experiment was in a commercial sugarcane growing region, characterised with mild winter, hot summer and a summer‐dominant rainfall. Weather data was recorded for the duration of crop growth using an automatic weather station located less than 200 m from the experimental site. Daily maximum and minimum temperature, and daily average radiation and rainfall during the trial period are reported in Figure 1. Temperature and radiation had an upward trend from June 2013 to January 2014 and a general downward trend from thereon with the beginning of the wet season. Mean temperature and radiation during the crop cycle were 29.3°C and 19.4 MJ m^−2^, respectively, which were close to long‐term climate statistics for the region.

Twenty sugarcane genotypes, contrasting for yield under water‐limiting conditions and representing a broad genetic population were planted in June 2013 (Table 1). The population comprised of commercial genotypes (selected for cane yield, sugar content and disease resistance in the Australian sugarcane breeding programme), unreleased but promising genotypes (advanced clones), and introgression clones that have not been subject to selection for cane or sugar yield. Three replicates of each genotype were planted in 10 m long 4 row plots with a row spacing of 1.5 m. The plots were subject to two levels of water treatment, rainfed (RF) and irrigated (IR). The plots were arranged in a split plot design with irrigation applied as main‐plot treatment and genotypes as subplots. Pest, pathogen, and nutrients were managed according to the Australian sugar industry best management practices.

**TABLE 1:** Classification and parents of sugarcane genotypes included in the field experiment

Genotype	Female parent	Male parent	Classification
KQ228	QN80‐3425	CP74‐2005	Commercial cultivar
MQ239	Q96	MQ77‐340	Commercial cultivar
N29	70E457	CP57‐614	Commercial cultivar
Q183	Q124	H56‐752	Commercial cultivar
Q208	Q135	QN61‐1232	Commercial cultivar
Q229	QN81‐289	QC75‐326	Commercial cultivar
Q240	QN81‐289	SP78‐3137	Commercial cultivar
Q252	Q208	Q96	Commercial cultivar
Q256	N21	Q135	Commercial cultivar
QA01‐5267	QA93‐2768	QA94‐6003	Advanced clone
QA04‐1448	QN80‐4316	Q173	Advanced clone
QB01‐10005	POJ2878	MANDALAY	Introgression
QBYC05‐20735	HoCP94‐806	Yacheng96‐29	Introgression
QBYC05‐20853	Neijiang57‐416	Yacheng96‐27	Introgression
QC91‐580	QN83‐636	Q142	Advanced clone
QN04‐121	QN89‐181	QN89‐109	Advanced clone
QN04‐1643	ARRIS	Q172	Advanced clone
QN66‐2008	EROS	QB64‐33	Advanced clone
QS00‐486	QN85‐1271	QN86‐2168	Advanced clone
QS01‐1078	68 W1049	QS88‐6007	Advanced clone

### 2.2 Irrigation treatment and stress index

**FIGURE 2:**
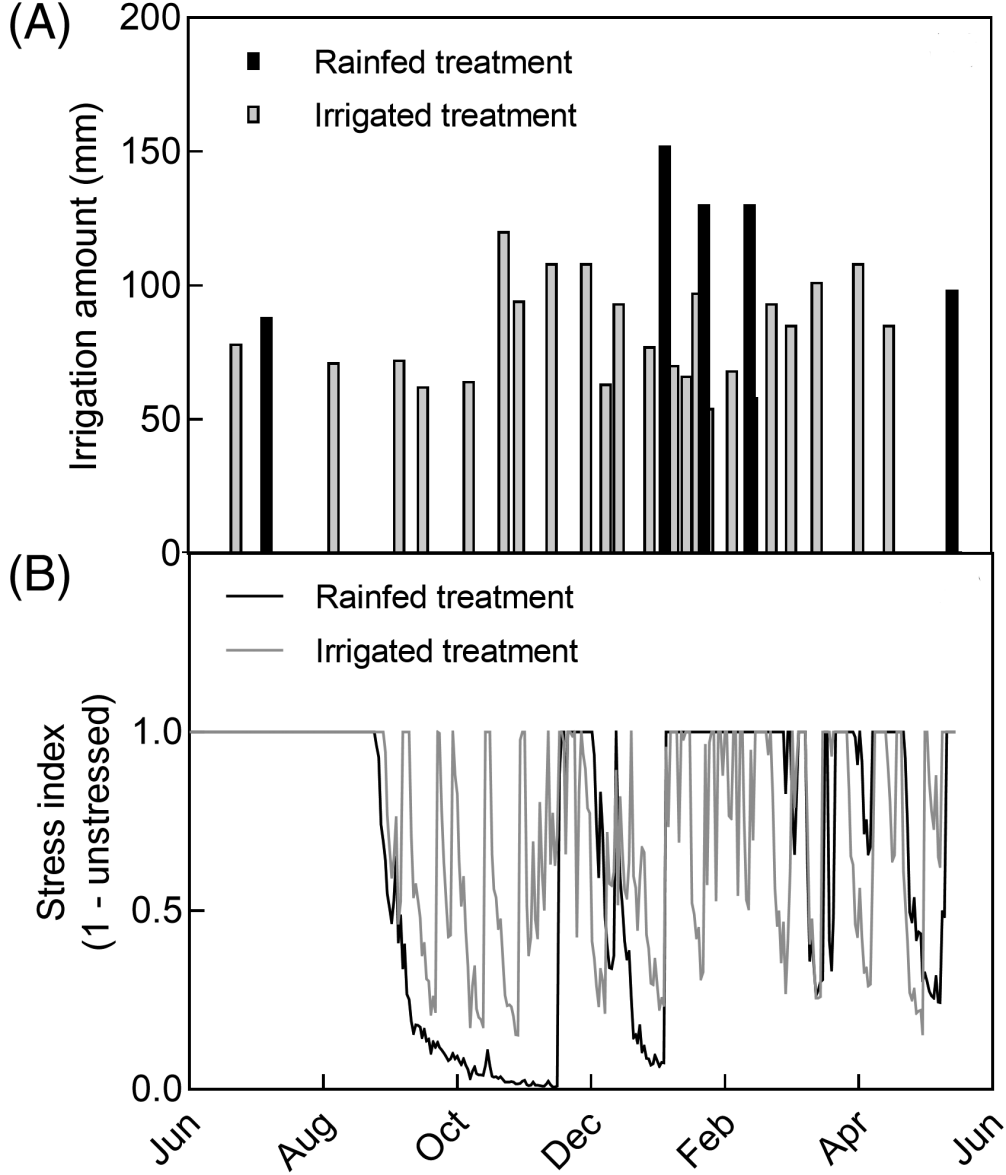
Irrigation amounts applied in rainfed and irrigated treatments (A); and APSIM simulated stress levels in rainfed and irrigated treatments (B). A stress index of 1 implies no water stress and 0 severe water stress

The irrigated plots were watered approximately at fortnightly intervals with an average 80 mm water per irrigation. Irrigation was withheld from the rainfed treatment 48 days after planting (DAP) (7 July 2013) when the crop was fully established until 6 January 2014 when it was watered to keep the crop alive (Figure 2A).

APSIM v7.5 (Keating *et al*., 2003) was used to simulate soil water stress index during the growing season. APSIM sugar model with the default variety Q117 and a clay over coarse sandy soil from Herbert and Burdekin region that closely resembles the soil physical properties of the field experiment were used as simulation parameters. The stress index (SI) was calculated as the ratio of plant available soil water supply to the plant transpiration demand. A stress index of 1 implies no stress and 0 indicates high stress. If the water supply from the soil profile exceeds crop water demand, then the required water is removed from the root‐occupied soil layers in proportion. If supply from the profile is less than the demand, then stress index declines in proportion.

As shown in Figure 2B, the rainfed treatment experienced incremental water stress from September to November 2013. This period of water stress was interrupted by a rainfall of 66 mm received from 18 to 24 November 2013. After this rainfall event, water stress in the rainfed treatment continued to increase until the rainfed crop was irrigated on 6 January 2014 to avoid terminal crop loss. There was sporadic rainfall in the following months until harvest in May 2014 (Figure 1). During the entire crop season, the irrigated treatment received about 20 megalitres (Ml) of water and the rainfed treatment received approximately 6 Ml. The relatively dry period from August to November 2013 meant the water stress imposed was severe with an average simulated stress index of 0.3 in the rainfed treatment. The irrigated treatment plots had a stress index of 0.8 in the same period (Figure 2B).

### 2.3 Early stage agronomic measurements

Early stage agronomic measurements were carried out on 19 November 2014, immediately following the first rainfall event to avoid potential loss of irrigation treatment effects. Therefore, the early stage represents the dry growth period before start of the wet season. The number of stalks at early stage in the second and third rows of each plot was counted manually and expressed as stalk number per metre (ESN). Early stage TDM (ETDM) was measured in all plots by randomly sub‐sampling eight stalks per plot, four each from the second and third rows. Leaf and stem portions were separated and dried in an oven at 60°C until constant weight. Finally, ETDM was calculated as the product of total leaf and stem dry weights and ESN. Early stage leaf area (ELA) of all leaves in a sub‐sample of three stalks was measured using a leaf area metre (LI‐3100C Area Meter, LI‐COR Biosciences).

### 2.4 Agronomic measurements at harvest

The field experiment was mechanically harvested on 12 May 2014 and the middle two rows of each experimental plot were weighed to determine TCH. Just before harvest, eight randomly selected stalks were sub‐sampled from the middle two rows, and leaf area (LA) and leaf dry weight were determined following the same procedure described above in the early stage. From the stem portions, sugarcane juice was extracted using a high‐pressure (355 kg cm^−1^) laboratory press and the remaining stem residue was weighed and dried in an oven at 60°C to a constant weight to determine fibre content. The juice extract was analysed using a polarimeter and a UV–VIS spectrophotometer to estimate total soluble solids and sugar content according to the Laboratory Manual for Australian Sugar Mills (BSES, 2001). Leaf dry weight, fibre content and the total soluble solids in the juice accounted for TDM (Basnayake *et al*., 2012).

### 2.5 Leaf‐level physiological measurements

Leaf gas exchange was measured on the sunlit part of the youngest fully expanded leaf on two tagged plants in all three replicates. Measurements were carried out on 2 days in each treatment in October 2013 (RF: 2 and 28 October and IR: 3 and 29 October) between 10 AM and 2:30 PM on each day. Leaf *A*, *g*_s_ and *C*_i_ were measured using an infrared gas analyser (IRGA) and portable photosynthesis system (LI‐6400XT, Li‐COR Biosciences). CO_2_ concentration was maintained at 400 μmol mol^−1^, photosynthetic active radiation was set to saturated light conditions of 2000 μmol m^−2^ s^−1^ and block temperature of the IRGA was set to 2°C above the ambient temperature. Leaves were allowed to acclimate in the gas analyser leaf cuvette until *A* and *g*_s_ were stable, i.e. when standard deviation in flow rate, *A* and *g*_s_ were less than 5 μmol s^−1^, 1 μmol m^−2^ s^−1^ and 0.1  mol m^−2^ s^−1^, respectively, for 15 s. Intrinsic transpiration efficiency was calculated as the ratio of *A* and *g*_s_.

### 2.6 Statistical analysis

#### 2.6.1 Spatial adjustment

Spatial analysis of the agronomic traits in each treatment was performed using a two‐dimensional penalised splines (P‐splines) ANOVA (PS‐ANOVA) surface smoothing model, referred as the SpATS model (Rodríguez‐Álvarez *et al*., 2018). The model was implemented using the freely available SpATS package (Rodríguez‐Álvarez *et al*., 2018) in R (R Foundation for Statistical Computing). The model default parameters of cubic B‐splines and second order penalties were used. The number of segments was set to 15, 4 in row and column direction, respectively. Spatially adjusted values of the agronomic traits were used for further statistical analysis.

#### 2.6.2 Variance components, heritability, and genetic correlation

Individual plot measurements of agronomic traits and average plot values of physiological traits were used for analysis of variances and covariances. The physiological traits measured on different days were analysed for genotype × date (G × D) interaction initially, followed by analysis of individual days. Variance and covariances of traits were estimated, considering genotypes as random effects, based on the REML method using the MIXED procedure in SAS (Version 9.4, SAS Institute). The following model was used for analysis within individual treatment:

$$Y_{\mathrm{ij}}=\mu +{\mathrm{Rep}}_{i}+{\mathrm{Gen}}_{j}+e_{\mathrm{ij}},$$

where μ is the mean, Rep_*i*_ is the effect of *i* ^th^ replicate, Gen_*j*_ is the effect of the *j* ^th^ genotype, and e_*ij*_ is the residual error. Heritability within single treatment was calculated as.

$$H_{b}^{2}=\frac{\sigma_{g}^{2}}{\sigma_{g}^{2}+\sigma_{\frac{e}{n_{r}}}^{2}}$$

where σ^2^_*g*_ and σ^2^_*e*_ are genetic and error variances, and *n*_*r*_ is the number of replications.

The model used for combined analysis across treatments was:

$$Y_{\mathrm{ijk}}=\mu +{\mathrm{Trt}}_{i}+{\mathrm{Rep}}_{j}\;\left({\mathrm{Trt}}_{i}\right)+{\mathrm{Gen}}_{k}+{\mathrm{Trt}}_{i}\times {\mathrm{Gen}}_{k}+e_{\mathrm{ijk}},$$

where μ is the mean, Trt_*i*_ and Trt_*i*_ × Gen_*k*_ are irrigation treatment and G × T effects, respectively. Heritability of combined treatments was estimated as:

$$H_{b}^{2}=\frac{\sigma_{g}^{2}}{\sigma_{g}^{2}+\sigma_{\frac{\mathrm{ge}}{n_{e}}}^{2}+\sigma_{\frac{e}{n_{e}n_{r}}}^{2}},$$

where σ^2^_*ge*_ is the G × T variance and error variance, respectively, and *n*_*e*_ is the number of treatments.

Phenotypic correlation between traits was determined on genotype means using Pearson correlation coefficient. Genetic correlations between traits were estimated using:

$$\gamma_{g}=\frac{\;{\mathrm{Cov}}_{g\left(\mathrm{xy}\right)}}{\sqrt{\sigma_{\mathrm{gx}}^{2}}\times \sqrt{\sigma_{\mathrm{gy}}^{2}}},$$

where Cov_*g*(*xy*)_ is the genetic covariance between two traits, and σ^2^_*gx*_ and σ^2^_*gy*_ are the genetic variance of the two traits.

#### 2.6.3 Regression analysis of physiological traits

Relationship between pairs of individual observations of physiological traits was analysed using linear or non‐linear regression and the best fit model was reported. To determine the break point *g*_s_ at which non‐stomatal limitations precede stomatal limitation, a segmented (piecewise) regression was employed in the ‘segmented’ R package (Muggeo, 2008).

#### 2.6.4 Variation in photosynthesis independent of stomatal conductance

To separate component traits of _i_ *TE* and to investigate variation in *A*_c_ independent of *g*_s_, differences in *A* at a reference *g*_s_ were determined (Gilbert *et al*., 2011). The relationship between *A* and *g*_s_ is generally non‐linear. This was transformed to a linear function using natural logarithm (ln) of *g*_s_. An analysis of covariance (ANCOVA) was carried out with individual observations of *A* as the response variable, transformed *g*_s_ as the covariate and with genotype as a classification factor. Initially, the interaction term genotype × ln(*g*_s_) was included in the ANCOVA model to test the assumption of equal slopes. If the slopes were not significant, the interaction term was removed from the ANCOVA model.

## 3 RESULTS

### 3.1 Irrigation treatment and genotypic effects on agronomic traits

Among early stage agronomic traits in the rainfed treatment, ESN and ELA had high genotypic variance and heritability, whereas ETDM had lower heritability (Table 2). The genotypes QN04‐1643 and Q208 had high ETDM, while QS00‐486 and QB01‐10005 had low ETDM. In irrigated conditions, all traits had high heritability, Q240 had the largest ETDM and Q183 had the largest ELA. The introgression genotype QB01‐10005 had the highest ESN in both irrigated and rainfed treatment conditions. Reduction of ETDM in the rainfed treatment was largest in Q240 (73%) and smallest in QS01‐1078 (54%). When combined across irrigation treatments, G × T variance was larger than genotype variance for ETDM and ELA, but not for ESN.

**TABLE 2:** Mean, variance components and heritability of early stage total dry matter (ETDM t ha^−1^), green leaf area per stalk (ELA; m^2^) and stalk number per metre (ESN) and harvest stage total dry matter (TDM; t ha^−1^), tonnes of cane yield per hectare (TCH; t ha^−1^), green leaf area (LA; m^2^) and stalk number per metre (SN) of 20 genotypes in two irrigation treatments

	ETDM		ELA		ESN		TDM		TCH		LA		SN	
	RF	IR	RF	IR	RF	IR	RF	IR	RF	IR	RF	IR	RF	IR
KQ228	2.96	9.95	3.64	10.92	14.09	16.34	27.05	56.15	69.62	154.43	26.46	41.23	13.96	13.24
MQ239	3.61	11.82	4.25	12.00	15.52	17.62	24.89	49.67	71.94	147.89	38.72	41.11	11.51	11.74
N29	4.01	9.02	3.36	9.64	19.31	16.73	22.19	48.10	54.75	137.02	33.39	32.03	12.98	14.87
Q183	3.93	11.29	3.52	16.72	18.25	13.81	18.20	46.03	52.85	133.54	29.66	52.79	8.95	10.95
Q208	4.37	10.49	4.46	9.65	17.51	17.16	24.25	41.79	68.14	141.03	23.67	27.89	13.25	14.83
Q229	3.80	9.92	3.32	9.94	17.14	19.47	23.49	52.32	64.87	158.65	29.22	35.46	12.36	14.66
Q240	3.91	14.60	3.73	12.59	18.09	17.54	19.51	55.03	58.33	165.09	35.01	33.08	8.75	12.31
Q252	3.52	11.15	4.42	10.64	14.56	16.20	21.17	51.53	62.23	161.71	27.15	29.84	10.28	15.01
Q256	3.38	8.96	3.90	9.48	16.37	17.90	25.28	52.87	73.63	162.98	32.03	33.49	12.25	17.47
QA01‐5267	2.95	8.05	3.18	9.79	11.86	13.32	16.42	44.48	46.31	140.19	37.91	39.16	7.41	13.48
QA04‐1448	3.23	9.25	4.47	12.04	13.63	15.40	23.82	55.70	75.53	192.62	40.38	42.83	10.50	13.26
QB01‐10005	2.87	9.54	2.10	9.65	22.60	21.46	12.03	33.34	30.06	91.77	17.41	30.74	16.95	16.83
QBYC05‐20735	4.12	9.31	3.28	9.22	18.82	18.44	23.87	48.60	61.71	143.35	30.19	29.59	12.03	14.25
QBYC05‐20853	3.86	10.56	4.49	10.09	15.84	19.93	20.40	39.23	50.63	109.92	34.15	26.67	11.25	12.71
QC91‐580	3.54	10.54	3.74	10.06	16.18	19.68	17.92	50.20	51.69	156.22	36.19	26.82	10.39	17.36
QN04‐121	2.95	6.83	3.54	10.19	13.58	13.83	19.39	44.89	58.12	137.55	34.75	40.69	9.37	10.51
QN04‐1643	4.49	11.04	3.88	10.45	22.17	19.18	18.63	50.86	55.06	167.40	42.05	26.79	8.72	17.19
QN66‐2008	3.76	11.72	3.61	11.83	13.19	15.94	20.69	47.28	64.66	149.89	42.15	40.62	9.82	13.70
QS00‐486	2.78	8.01	2.82	8.31	16.77	20.14	25.78	48.80	69.41	151.27	29.61	29.99	14.62	16.41
QS01‐1078	3.59	7.97	2.79	9.00	21.18	18.62	21.13	37.74	57.59	123.84	30.61	16.03	11.83	15.82
Mean	3.58	10.00	3.62	10.61	16.83	17.44	21.31	47.73	59.86	146.32	32.53	33.84	11.36	14.33
G	0.14	2.77	0.34	3.24	8.2	4.94	11.89	34.75	99.78	443.11	37.83	63.72	4.97	4.18
Residual	0.34	0.77	0.17	0.18	2.23	0.56	4.62	7.49	52.06	94.31	4.91	4.11	1.16	0.76
Heritability	0.55	0.91	0.86	0.98	0.92	0.96	0.89	0.93	0.85	0.93	0.96	0.98	0.93	0.94
Overall Mean	6.79		7.12		17.13		34.52		103.09		33.19		12.84	
G	0.45		0.32		4.15		12.38		168.58		9.93		2.02	
G × T	1.01		1.47		2.42		10.94		102.86		40.84		2.55	
Residual	0.56		0.17		1.40		6.05		73.19		4.51		0.96	
Heritability	0.43		0.29		0.74		0.66		0.73		0.32		0.58	

Abbreviations: G, genotype variance; G × T, genotype × irrigation treatment variance; IR, irrigated treatment; RF, rainfed treatment.

Genotypic variance and heritability were high for all traits measured at harvest within irrigation treatment (Table 2). Total dry matter varied from 12.03 to 27.05 t ha^−1^ in the rainfed treatment and from 33.34 to 56.15 t ha^−1^ in the irrigated treatment, while the range in TCH was 30.06 to 75.53 t ha^−1^ in the rainfed and 91.77 to 192.62 t ha^−1^ in the irrigated treatment. Reduction due to water stress ranged from 41 to 64% and 51 to 67% for TDM and TCH, respectively. The reduction was smallest in Q208 and largest in QB01‐10005. Mean SN was higher in the irrigated treatment than in the rainfed treatment, whereas some genotypes such as QS01‐1078 and QC91‐580 had higher leaf area in the rainfed treatment than in the irrigated treatment. G × T was smaller than genotype variance for TDM and TCH, and larger than genotype variance for LA and SN. Irrespective of the irrigation treatment, commercial genotypes had higher mean TDM and TCH than advanced and introgression clones.

Stalk number per metre measured at early stage and during harvest had a significant correlation in both rainfed and irrigated conditions (Table 3). Leaf area at both stages of measurement had significant correlation in the irrigated treatment. However, other agronomic traits measured at early stage had poor correlation with the same traits measured at harvest.

**TABLE 3:** Phenotypic (lower diagonal) and genetic correlation (upper diagonal) between agronomic traits measured at early and harvest stages

	ETDM	ELA	ESN	TDM	TCH	LA	SN
Rainfed treatment							
ETDM	1	0.39 ns	0.36 ns	−0.07 ns	−0.05 ns	0.22 ns	−0.44 ns
ELA	0.42 ns	1	−0.53*	0.59**	0.42 ns	0.42 ns	−0.46*
ESN	0.38 ns	−0.45*	1	−0.56*	−0.39 ns	−0.42 ns	0.37 ns
TDM	0.14 ns	0.55*	−0.39 ns	1	0.9***	0.28 ns	−0.12 ns
TCH	0.2 ns	0.43 ns	−0.23 ns	0.92***	1	0.04 ns	0.17 ns
LA	0.14 ns	0.4 ns	−0.44 ns	0.33 ns	0.15 ns	1	−0.78***
SN	−0.2 ns	−0.42 ns	0.47*	−0.08 ns	0.15 ns	−0.71***	1
Irrigated treatment							
ETDM	1	0.56**	0.06 ns	0.21 ns	0.32 ns	0.13 ns	−0.2 ns
ELA	0.56**	1	−0.53*	0.17 ns	0.25 ns	0.74***	−0.63**
ESN	0.1 ns	−0.5*	1	−0.31 ns	−0.27 ns	−0.69***	0.67**
TDM	0.22 ns	0.16 ns	−0.26 ns	1	0.9***	0.24 ns	0 ns
TCH	0.33 ns	0.23 ns	−0.21 ns	0.93***	1	0.37 ns	−0.12 ns
LA	0.13 ns	0.74***	−0.67**	0.22 ns	0.31 ns	1	−0.7***
SN	−0.18 ns	−0.61**	0.65**	0.04 ns	−0.04 ns	−0.65**	1

Abbreviations: ELA, early green leaf area per stalk; ESN, early stage stalk number per metre; ETDM, early stage total dry matter; LA, green leaf area at maturity; ns, not significant; SN, stalk number per metre; TCH, cane yield per hectare; TDM, harvest stage total dry matter.

*p < 0.05; **p < 0.01; ***p < 0.001.

### 3.2 Irrigation treatment and genotypic effects on leaf‐level physiological traits

Water stress had significant effect on *A* and *g*_s_ causing a sharp decline in both attributes (Table 4). Mean reduction in *A* and *g*_s_ due to water stress was 56 and 62%, respectively. QS01‐1078 had the smallest reduction, while QB01‐10005 and Q240 had the largest reduction in *A* and *g*_s_ due to water stress. Genotypes showed diverse response in _i_ *TE*, for instance, a 37% increase in _i_ *TE* was observed in QB01‐10005 whereas a 7% decline was recoded for Q252 due to water stress. Photosynthesis and *g*_s_ showed moderate‐high heritability in both treatments (Table 4). Genotypic variation for _i_ *TE* was significant in the irrigated treatment but not in the rainfed treatment. When data was pooled across the 2 days of measurement and irrigation treatments, *A* and *g*_s_ had high variance associated with the irrigation treatment. Variance in _i_ *TE* was mostly due to the measurement days along with a large residual variance, as there was no variance associated with the treatment effect. For other traits, variance components associated with G × D and G × T were smaller than main effects.

**TABLE 4:** Mean photosynthesis (*A;* μmol m^−2^ s^−1^), stomatal conductance (*g*_s_; mol m^−2^ s^−1^) and intrinsic transpiration efficiency (_i_ *TE;* μmol mol^−1^) of 20 genotypes measured in rainfed (RF) and irrigated (IR) treatments

Genotype	*A*		*g*_s_		_i_ *TE*	
	RF	IR	RF	IR	RF	IR
KQ228	14.05	39.84	0.10	0.37	135.59	107.32
MQ239	13.61	38.39	0.12	0.38	122.26	106.81
N29	18.28	40.38	0.13	0.32	142.74	131.82
Q183	16.23	34.43	0.12	0.29	132.98	126.94
Q208	12.07	30.78	0.08	0.31	137.05	111.79
Q229	14.08	45.85	0.11	0.46	118.96	104.58
Q240	8.61	41.15	0.07	0.36	114.76	117.08
Q252	15.80	34.73	0.12	0.26	130.82	137.13
Q256	14.27	38.30	0.10	0.30	138.42	132.00
QA01‐5267	22.51	36.10	0.20	0.35	116.84	116.13
QA04‐1448	14.17	31.60	0.09	0.25	151.27	126.53
QB01‐10005	12.55	42.28	0.10	0.56	113.04	79.02
QBYC05‐20735	15.05	35.73	0.12	0.28	137.98	135.97
QBYC05‐20853	21.90	42.59	0.20	0.41	123.49	104.36
QC91‐580	19.64	33.58	0.16	0.29	141.36	122.30
QN04‐121	22.36	38.15	0.20	0.51	118.56	84.07
QN04‐1643	18.11	28.51	0.13	0.20	144.80	151.88
QN66‐2008	15.32	36.24	0.13	0.34	122.44	115.12
QS00‐486	14.64	38.09	0.11	0.32	135.87	123.61
QS01‐1078	26.31	36.78	0.20	0.33	132.67	115.12
Mean	16.44	37.1	0.13	0.34	130.91	118.84
G	10.36**	11.36**	0.001**	0.004**	54.78 ns	189.8**
Residual	21.28	18.35	0.001	0.006	310.52	276.58
Heritability	0.59	0.65	0.66	0.69	0.35	0.67
Overall mean	27.47		0.24		123.55	
G, *n* (%)	12.4 (5)		0.003 (9)		106.1 (9)	
D, *n* (%)	25.5 (9)		0.001 (4)		358.3 (28)	
T, *n* (%)	199.4 (74)		0.022 (65)		0 (0)	
G × D, *n* (%)	1.5 (1)		0 (0)		50.9 (4)	
G × T, *n* (%)	0 (0)		0.001 (2)		0 (0)	
Residual, *n* (%)	30 (11)		0.007 (20)		751.3 (59)	

Abbreviations: D, date variance; G × D, genotype × date interaction variance; G × T, genotype × treatment interaction variance; G, genotype variance; ns, not significant; T, treatment variance.

*p < 0.05; **p < 0.01; ***p < 0.001.

**FIGURE 3:**
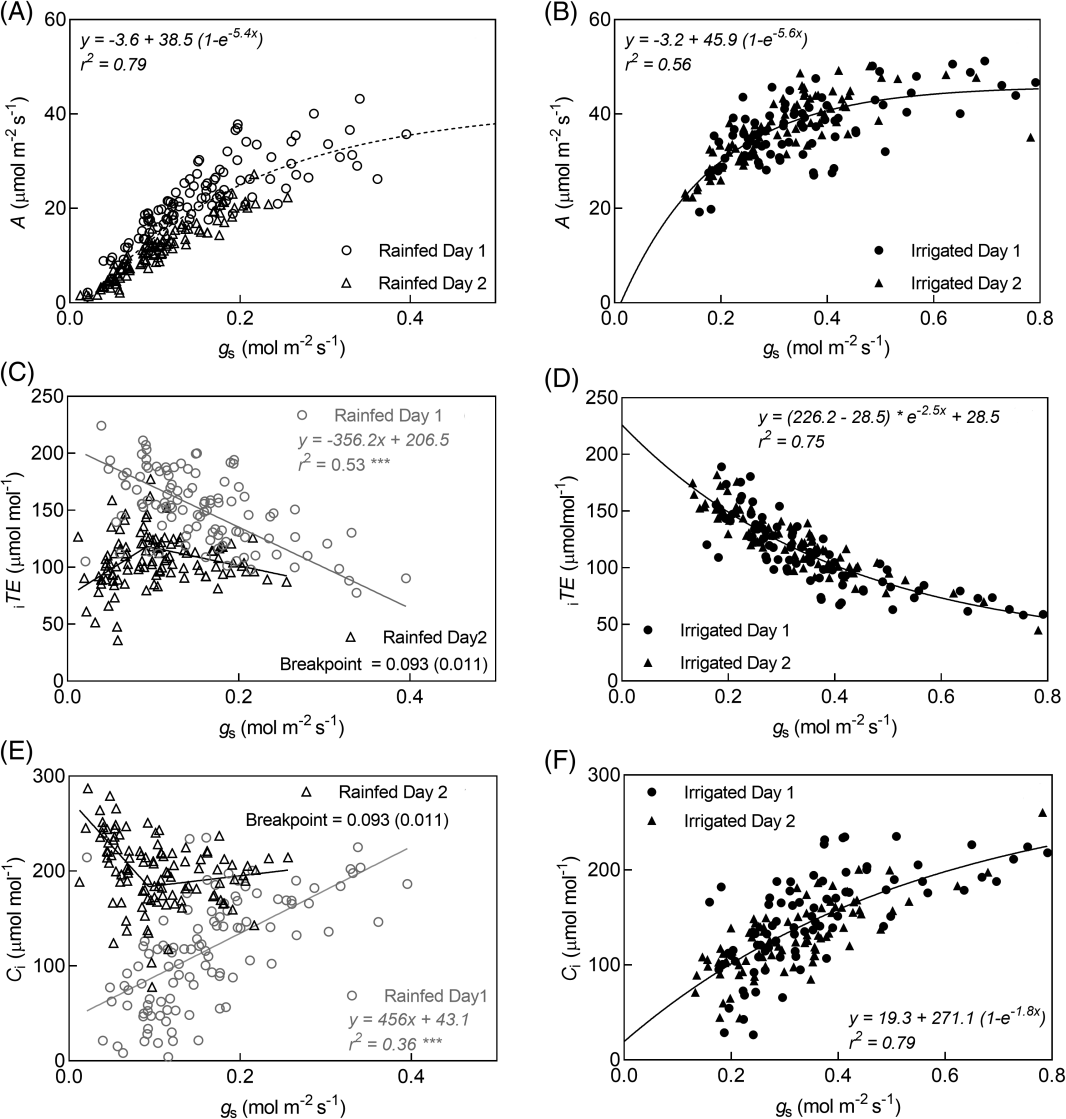
Non‐linear relationship between photosynthesis (*A*) and stomatal conductance (*g*_s_) in rainfed and irrigated treatments, respectively (A, B); linear regression of *g*_s_ and intrinsic transpiration efficiency (_i_ *TE*) in the rainfed treatment on day 1 and segmented regression of *g*_s_ and _i_ *TE* in the rainfed treatment on day 2 showing breakpoint *g*_s_ and standard error in parenthesis for the onset of non‐stomatal limitation (C); relationship between _i_ *TE* and *g*_s_ in irrigated treatment (D); linear regression of *g*_s_ and intercellular CO_2_ (*C*_i_) in the rainfed treatment on day 1 and segmented regression of *g*_s_ and *C*_i_ in the rainfed treatment on day 2 showing breakpoint *g*_s_ and standard error in parenthesis for the onset of non‐stomatal limitation (E); relationship between *C*_i_ and *g*_s_ in the irrigated treatment (F)

Stress index on gas exchange measurement days were 0.08 and 0.03 in rainfed and 0.8 and 0.5 in irrigated treatments (Figure 2B). Genotype variance was significant for all traits on all days except for *A* on Day 1 (Table 5). Photosynthesis and *g*_s_ had a non‐linear relationship with higher gains in *A* at low *g*_s_ than at high *g*_s_ levels (Figure 3A,B). In the irrigated treatment, mean *A* on both days of measurement was similar; while *g*_s_ was reduced on Day 2, which led to an increase in _i_ *TE* on Day 2. However, in the rainfed treatment, despite the reduced *g*_s_ on Day 2, _i_ *TE* declined (Table 5) at *g*_s_ below 0.09 mol m^−2^ s^−1^ (Figure 3C). Non‐stomatal limitation to *A* was evident at *g*_s_ <0.09 mol m^−2^ s^−1^ demonstrated by the low *A* despite high *C*_i_ (Figure 3E).

**TABLE 5:** Photosynthesis (*A;* μmol m^−2^ s^−1^), conductance (*g*_s_; mol m^−2^ s^−1^) and intrinsic transpiration efficiency (_i_ *TE;* μmol mol^−1^) separated by day of measurement in rainfed (RF) and irrigated (IR) treatments

	*A*		*g*_s_		_i_ *TE*	
	RF	IR	RF	IR	RF	IR
Day 1 stress index: RF = 0.08, IR = 0.8						
Mean	20.87	37.57	0.14	0.37	155.44	110.11
G	2.14 ns	0 ns	0.001*	0.005**	247.33***	252.67**
Residual	40.06	49.3	0.003	0.014	245.67	660.79
Heritability	0.14	0	0.54	0.55	0.75	0.53
Day 2 stress index: RF = 0.03, IR = 0.5						
Mean	12.08	36.9	0.11	0.32	108.14	123.25
G	18.8***	25.78***	0.001**	0.006***	143**	149.56*
Residual	9.49	11.4	0.001	0.002	182.38	181.12
Heritability	0.85	0.82	0.77	0.86	0.7	0.62

Abbreviation: ns, not significant.

*p < 0.05; **p < 0.01; ***p < 0.001.

### 3.3 Relationships between agronomic and physiological traits

Combined across days, mean _i_ *TE* of genotypes showed significant phenotypic and genetic correlation with TDM and TCH in the irrigated treatment (Table 6). On Day 1, TDM and TCH had a significant and positive phenotypic and genetic correlation with mean _i_ *TE* in both rainfed and irrigated treatments. Whereas on Day 2 the correlations were significant in irrigated but not in the rainfed treatment. Photosynthesis and *g*_s,_ when significant, had a negative correlation with TDM and TCH in both treatments.

**TABLE 6:** Phenotypic and genetic correlation between early total dry matter (ETDM; t ha^−1^), total dry matter at harvest (TDM; t ha^−1^), tonnes cane per hectare at harvest (TCH; t ha^−1^) and photosynthesis (*A;* μmol m^−2^ s^−1^), stomatal conductance (*g*_s_; mol m^−2^ s^−1^) and intrinsic transpiration efficiency (_i_ *TE;* μmol mol^−1^) in rainfed (RF) and irrigated (IR) treatments

		*A*		*g*_s_		_i_ *TE*	
		RF	IR	RF	IR	RF	IR
Overall	Phenotypic correlation						
	ETDM	−0.21 ns	−0.06 ns	−0.34 ns	−0.26 ns	0.39 ns	0.2 ns
	TDM	−0.21 ns	−0.16 ns	−0.24 ns	−0.44 ns	0.27 ns	0.5*
	TCH	−0.27 ns	−0.38 ns	−0.29 ns	−0.56*	0.25 ns	0.54*
	Genotypic correlation						
	ETDM	−0.42 ns	−0.08 ns	−0.62**	−0.35 ns	na	0.3 ns
	TDM	−0.41 ns	−0.17 ns	−0.45*	−0.53*	na	0.58**
	TCH	−0.51*	−0.48*	−0.52*	−0.72***	na	0.72***
Day 1	Phenotypic correlation						
	ETDM	−0.13 ns	0 ns	−0.3 ns	−0.17 ns	0.44 ns	0.09 ns
	TDM	−0.17 ns	−0.23 ns	−0.35 ns	−0.49*	0.66**	0.45*
	TCH	−0.27 ns	−0.34 ns	−0.43 ns	−0.55*	0.71***	0.48*
	Genotypic correlation						
	ETDM	na	na	−0.59**	−0.36 ns	0.51*	0.11 ns
	TDM	na	na	−0.61**	−0.56*	0.88***	0.51*
	TCH	na	na	−0.74***	−0.7***	0.99***	0.62**
Day 2	Phenotypic correlation						
	ETDM	0.03 ns	0.03 ns	−0.01 ns	−0.17 ns	0.07 ns	0.24 ns
	TDM	−0.09 ns	−0.15 ns	−0.11 ns	−0.37 ns	0.15 ns	0.5*
	TCH	−0.22 ns	−0.41 ns	−0.23 ns	−0.55*	0.11 ns	0.56*
	Genotypic correlation						
	ETDM	−0.02 ns	0.03 ns	−0.13 ns	−0.28 ns	0.05 ns	0.44 ns
	TDM	−0.25 ns	−0.11 ns	−0.37 ns	−0.36 ns	0.27 ns	0.6**
	TCH	−0.42 ns	−0.44*	−0.56**	−0.63**	0.21 ns	0.8***

Abbreviations: na, not applicable; ns, not significant.

*p < 0.05; **p < 0.01; ***p < 0.001.

The correlation between _i_ *TE*, and TDM and TCH were further analysed after separating gas exchange observation from both irrigation treatments into two distinct *g*_s_ ranges, to filter out the non‐stomatal limitation region of *g*_s_ (<0.1 mol m^−2^ s^−1^). In *g*_s_ range between 0 and 0.1 mol m^−2^ s^−1^, genotypic variation in _i_ *TE* was not significant and was associated with large error variance (Table 7). In the *g*_s_ range between 0.1–0.4 mol m^−2^ s^−1^, _i_ *TE* was characterised with significant genotypic variance, high heritability and significant genetic correlation with ETDM, TDM and TCH.

**TABLE 7:** Intrinsic transpiration efficiency (_i_ *TE;* μmol mol^−1^) separated by stomatal conductance (*g*_s_; mol m^−2^ s^−1^) range, and its phenotypic and genetic correlation with early total dry matter (ETDM), total dry matter (TDM) and tonnes cane per hectare (TCH)

	*g*_s_ range (mol m^−2^ s^−1^)	
	0–0.1	0.1–0.4
Mean	138.82	124.69
Genotype variance	159.41 ns	102.92**
Residual variance	1744.50	177.58
Heritability	0.22	0.63
Phenotypic correlation		
ETDM	−0.25 ns	0.05 ns
TDM	0.11 ns	0.52*
TCH	0.07 ns	0.53*
Genetic correlation		
ETDM	na	0.32 ns
TDM	na	0.78***
TCH	na	0.75***

Abbreviations: na, not applicable; ns, not significant.

*p < 0.05; **p < 0.01; ***p < 0.001.

### 3.4 Genotypic variation in photosynthesis at reference conductance

**FIGURE 4:**
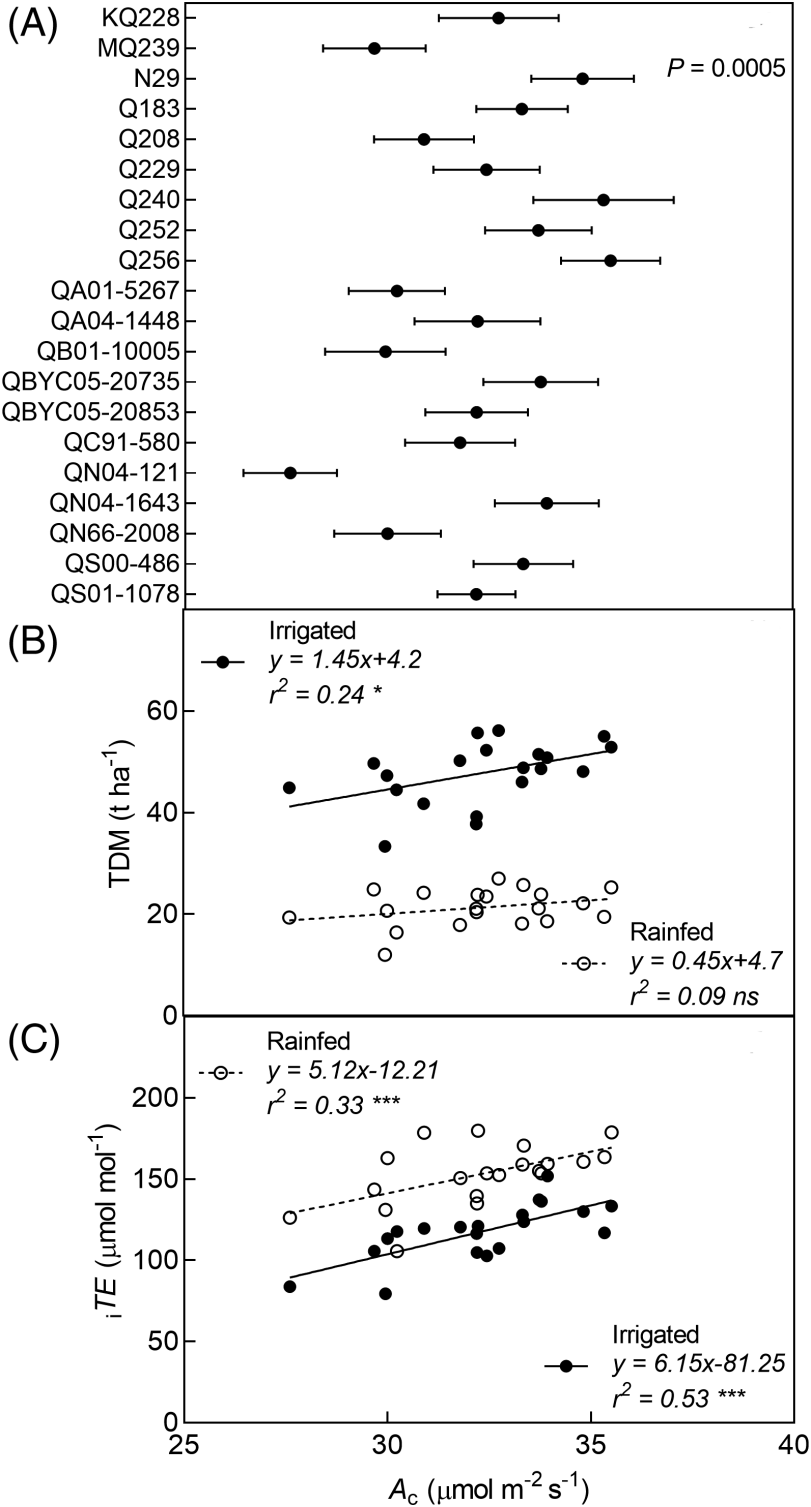
Mean photosynthetic capacity (*A*_c_) at reference stomatal conductance (*g*_s_) of 0.26 mol m^−2^ s^−1^ (A); relationship of *A*_c_ with total dry matter (TDM) (B); and intrinsic transpiration efficiency (_i_ *TE*) (C) of 20 genotypes in two water treatments. _i_ *TE* observations in day 2 rainfed treatment that showed non‐stomatal limitation to *A* were excluded. ns, not significant; *p < 0.05; **p < 0.01; ***p < 0.001

Gas exchange observations when *g*_s_ <0.1 mol m^−2^ s^−1^ that showed non‐stomatal limitations were excluded from this analysis. The non‐linear relationship between *A* and *g*_s_ for each genotype and the transformed linear relationship using ln(*g*_s_) is shown in the Figure S1. An ANCOVA model of ln(*g*_s_) and *A* with interaction term included showed no significant effect for the interaction term, indicating difference between the slopes of genotypes was not significant (Table S1). Therefore, *A* was analysed using a general ANCOVA model with transformed *g*_s_ as the covariate and genotype as a factor. The genotypic factor was significant in determining the response variable *A* (*F* = 1.06, *p* < 0.001) (Table S1), which shows that there are significant differences in *A* of genotypes. Figure 4A shows the mean *A*_c_ of genotypes at the average value of the covariate (*g*_s_ = 0.26 mol m^−2^ s^−1^). At the reference *g*_s_, Q240 and Q256 had high *A*_c_ while QN01‐121 had the lowest *A*_c_. From this analysis it could be concluded that *A*_c_ variation at a reference *g*_s_ was significant. It was also significantly correlated to TDM in the irrigated treatment and to _i_ *TE* in both rainfed and irrigated treatments (Figure 4B,C).

## 4 DISCUSSION

### 4.1 Genotype × irrigation treatment interaction not significant for cane yield

Genotypic variation was highly significant for TCH and TDM under rainfed and irrigated conditions. Overall, commercial genotypes had higher TCH and TDM than advanced, and introgression clones. This suggest that the performance of commercial genotypes selected for high yield in irrigated conditions was also maintained in rainfed conditions. Regardless of the large reduction in mean TCH and TDM due to water stress, by an average of 59 and 55%, respectively, G × T effect was not significant for either TCH or TDM. These results are in accordance with a previous study where cane yield of 10 genotypes in 24 environments including rainfed, irrigated and semi‐irrigated conditions showed little G × T (Natarajan *et al*., 2020). Similar results were reported earlier by Basnayake *et al*. (2012), where G × T interactions were small even when cane yield reduction was more than 50%. A possible explanation for the small G × T effect is that the yield was largely determined by growth under non‐limiting water conditions during the wet months even in the rainfed treatment. In this study, approximately 80% of the total TDM was accumulated during the wet and warm months from November to June in both irrigated and rainfed treatments. Furthermore, G × T variance component was larger than genotype variance component for ETDM (Table 2), however it was relatively smaller than genotype variance for TDM and TCH measured after the wet season. Therefore, irrespective of the early stage growing conditions, genotypes that were well‐adapted to growing under non‐limiting water conditions had higher cane yield in both irrigated and rainfed conditions.

### 4.2 Intrinsic transpiration efficiency a significant determinant of cane yield

Intrinsic transpiration efficiency showed significant correlation with TDM and TCH under both rainfed and irrigated conditions, indicating variation in _i_ *TE* was one of the mechanisms contributing to variation in TDM and TCH in this study. While modelling and pot‐based studies have identified _i_ *TE* as a valuable trait for improving sugarcane yield (Inman‐Bamber *et al*., 2012; Jackson *et al*., 2016; Stokes *et al*., 2016), this study validates the relationship between _i_ *TE* and cane yield under field conditions. The negative correlation between *g*_s_ and TDM or TCH observed in this study is contrary to a previous study in sugarcane that reported a wide range of genetic correlation (−0.29 to 0.94) between *g*_s_ and cane yield, and an overall positive relationship between the two (Basnayake *et al*., 2015). The disparity in genetic correlation in that study were attributed to a moderate correlation (*r* = −0.4) with ambient temperature on the day of measurement, with higher correlation observed on cooler days. Basnayake *et al*. (2015) measured *g*_s_ under ambient conditions using steady‐state porometer that can be confounded by environmental effects, whereas in this study leaves were exposed to a controlled environment IRGA chamber, which could be a possible reason for the different results obtained in the two studies. The results in this study show that *A* per unit *g*_s_, rather than *g*_s_ alone, is more important in determining cane yield under rainfed or irrigated conditions.

### 4.3 Genetic correlation between _i_*TE* and cane yield maximised under certain *g*_s_ ranges

At high *g*_s_, _i_ *TE* declined because *A* and *C*_i_ remained relatively stable while *g*_s_ continued to increase. This is because *A* in C4 plants reaches saturation levels at relatively low *C*_i_ and is insensitive to changes in *g*_s_ at higher levels (Ghannoum, 2009). While with declining *g*_s_, *A* and *C*_i_ decreased non‐linearly and _i_ *TE* increased because the decline in *A* rate were more gradual than decline in *C*_i_ rates. This is particularly true for C4 plants, which maintain *A* even at low *C*_i_ because of their superior carboxylation capacity (Von Caemmerer and Furbank, 2003). However, when *g*_s_ declined below 0.1 mol m^−2^ s^−1^ there were deviations from this trend with some observations characterised by high *C*_i_ and low _i_ *TE* (Figure 3C,E). Low *A* despite more than adequate *C*_i_ in the leaf indicates non‐stomatal inhibition of *A* at *g*_s_ below 0.1 mol m^−2^ s^−1^. Therefore, the *g*_s_ of 0.1 mol m^−2^ s^−1^ can be defined as the minimum functional *g*_s_ below which non‐stomatal limitations inhibit *A* (Brodribb, 1996). Inhibition of *A* due to biochemical limitation has been shown previously in four different C4 grasses when water stress reduced leaf relative water content to 40% (Ghannoum *et al*., 2003). In sugarcane, decline in pyruvate orthophosphate dikinase (PPDK) activity has been reported as the major non‐stomatal limitation in C4 photosynthesis when leaf water potential reduces below −0.85 MPa (Du *et al*., 1996).

Plants stomatal behaviour have evolved to optimise the trade‐off of CO_2_ uptake for *A* and transpiration water loss (Brodribb *et al*., 2009). At high *g*_s_ levels, rate of transpiration loss is greater than the assimilation gains and at low *g*_s_ levels non‐stomatal limitations can predominate stomatal limitations. In this study, measurements of _i_ *TE* when *g*_s_ <0.1 mol m^−2^ s^−1^ had high error variance compared to genotypic variance and thus had low heritability. Whereas _i_ *TE* examined in the *g*_s_ range between 0.1 and 0.4 mol m^−2^ s^−1^ showed significant genotypic variation and correlation with TDM and TCH. These results suggest that the genetic variation for _i_ *TE* and its correlation with crop productivity is maximised within specific *g*_s_ ranges. A similar result was reported earlier in pot‐grown sugarcane wherein *C*_i_ was related to whole‐plant *TE* but only when measured at a narrow *g*_s_ range (Jackson *et al*., 2016).

### 4.4 Understanding the components of _i_*TE* helps select high _i_*TE* genotypes for different production environments

**FIGURE 5:**
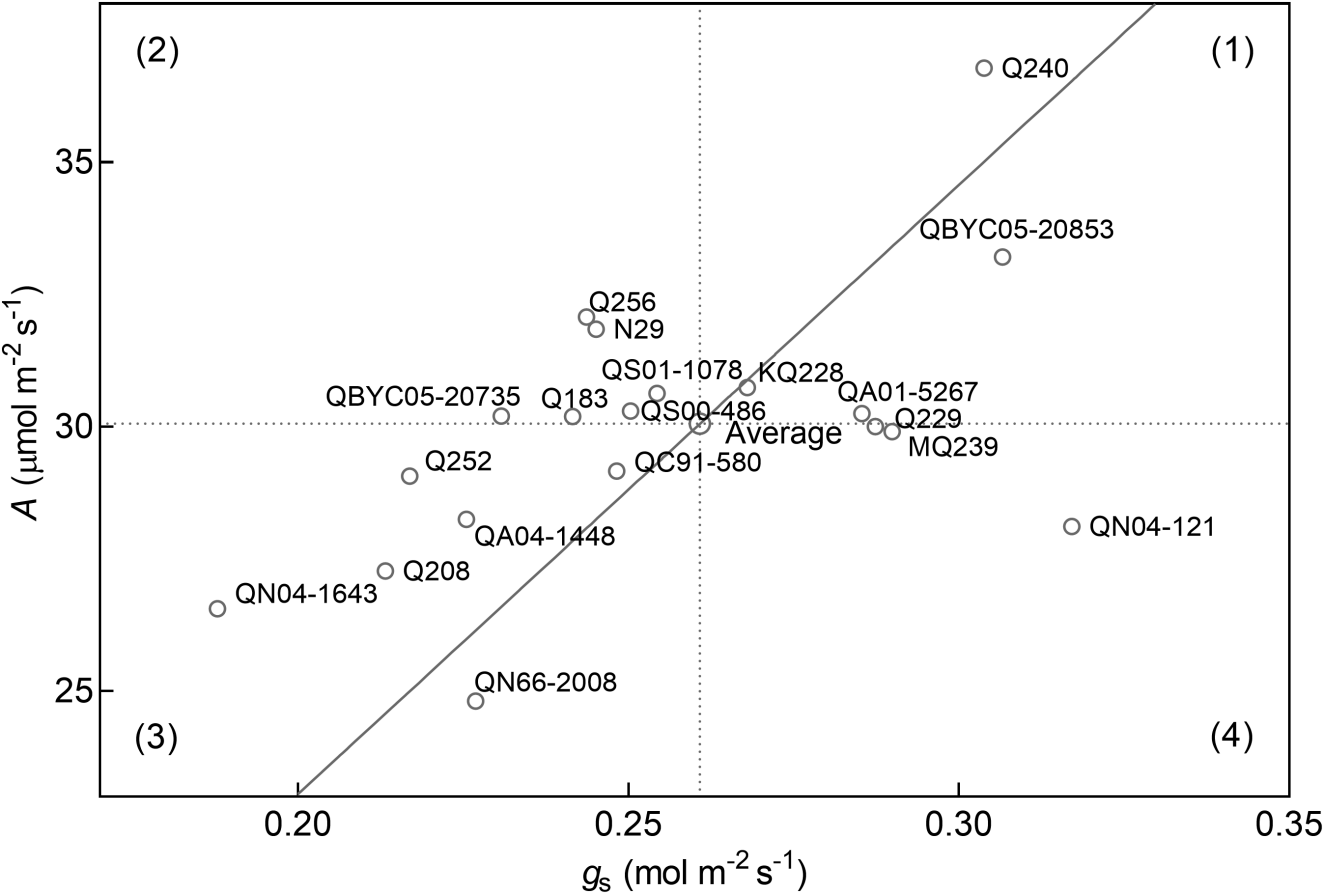
Average assimilation (*A*) and conductance (*g*_s_) of 20 genotypes relative to the population average *A* versus *g*_s_ regression fit. *g*_s_ observations below 0.1 mol m^−**2**^ s^−**1**^ that showed non‐stomatal limitation to *A* were excluded. All points on the diagonal line have _i_ *TE* equal to population average

Figure 5 is a scatterplot showing average *A* and *g*_s_ of each genotype when *g*_s_ >0.1 (mol m^−2^ s^−1^). The regression line represents the population average _i_ *TE*. Genotypes above and to the left of regression line have higher than average _i_ *TE*, and genotypes below and to the right of the line have lower than average _i_ *TE*. The plot area was divided into four quadrants, which shows all possible combinations of *A* and *g*_s_ of the genotypes to achieve the population average. Quadrant 1 represents genotypes with higher *A* and *g*_s_ relative to the average genotype. These genotypes could have potential for higher productivity in well‐watered environments. Genotypes within Quadrant 2, such as Q256 and N29, had higher *A* and lower *g*_s_ than the average genotype, which indicates they have the potential to increase productivity while using less water than the average genotype. Genotypes in Quadrant 3 had lower *g*_s_ and *A* compared to the average genotype that presumably will translate to conservative water use and productivity. Quadrant 4 represents genotypes with lower *A* and higher *g*_s_ relative to the average genotype, indicating inefficient use of water.

Figure 5 also provides vital insights into the adaptation of two of the most widely cultivated sugarcane genotypes in Australia. The Quadrant 1 genotype KQ228, which is only prevalent in irrigated production regions in Australia, had slightly higher than average *A* and *g*_s,_ and _i_ *TE* comparable to the average genotype. KQ228 would be well suited for environments with abundant water supply because it can maximise productivity by fully utilising available soil water, although inefficiently because of its average _i_ *TE*. On the other hand, the Quadrant 3 genotype Q208, which is the predominant variety in rainfed environments, had relatively low *A* and *g*_s_ than average but higher _i_ *TE* than average, indicating its suitability for environments with limited water supply because it has conservative water use characteristics and is efficient in utilising available water. Separation of _i_ *TE* into its components therefore provides important insights to determine if higher _i_ *TE* of genotypes will be beneficial for water conservation, overall productivity, or both.

There was significant difference in *A*_c_ independent of *g*_s_ (Figure 4A) and there was no interaction between transformed *g*_s_ and *A* (Table S1) indicating the differences in *A*_c_ between the genotypes were maintained for the range of *g*_s_ (0.1–0.4 mol^−1^ m^−2^ s^−1^) tested in this study. Genotypic differences in *A*_c_ were related to TDM in the irrigated treatment and to _i_ *TE* in both rainfed and irrigated treatments suggesting that *A*_c_ was at least partly responsible for differences in _i_ *TE* and TDM. Therefore, achieving high _i_ *TE* with high *A* is possible, and a potential strategy to overcome the trade‐off between high _i_ *TE* and low *g*_s_ would be the selection of genotypes with high _i_ *TE* and high *A*_c_ for any given *g*_s_ (Gilbert *et al*., 2011).

Despite the valuable genotypic variation and significant genetic correlation of _i_ *TE* with TDM and TCH shown in this study, characterising hundreds of genotypes typically evaluated in a breeding programme for _i_ *TE* using time‐consuming gas exchange measurements is not feasible. In addition, a practical issue experienced in this study, which may apply in general for sugarcane gas exchange measurements, is that the leaves at the top of canopy are inaccessible after the first few months of growth due to tallness of the crop. Measuring gas exchange at different levels of the canopy is complicated by the decoupling effects between leaf and the atmosphere, wherein it has been shown that the leaves at the base of a canopy are potentially more decoupled from the atmosphere than top canopy leaves (Jarvis and McNaughton, 1986). The rapidly advancing aerial field phenomics technologies currently being evaluated in sugarcane breeding (Natarajan *et al*., 2019) may offer solutions for the practical application of _i_ *TE* for variety development.

## 5 CONCLUSIONS

The value of _i_ *TE* in improving sugarcane productivity has been assessed theoretically in prior studies (Inman‐Bamber *et al*., 2012; Stokes *et al*., 2016). In this study, for the first time, the significant genotypic variation in _i_ *TE* and its genetic correlation with productivity in field conditions has been shown. Measuring _i_ *TE* under managed soil water conditions will be important because heritability of _i_ *TE* and its correlation with TDM and TCH was maximised in the *g*_s_ range between 0.1 and 0.4 mol m^−2^ s^−1^. Whereas under severe water stress, heritability of _i_ *TE* was low at *g*_s_ <0.1 mol m^−2^ s^−1^ with evidence of non‐stomatal limitation to *A*. The genotypes in this study also showed significant variation in *A*_c_ independent of *g*_s_, and it was related to _i_ *TE* in both treatment conditions and with TDM in irrigated condition_._ This shows that differences in _i_ *TE* of genotypes were at least partly due to *A*_c_, which suggests that there is potential for improving _i_ *TE* without the negative effects of low *g*_s_. Remote sensing of traits such as crop vigour, canopy spectral indices and canopy temperature as proxies for components of _i_ *TE* and incorporating them in a selection index (Natarajan *et al*., 2019) would be a practical approach for characterising sugarcane genotypes for _i_ *TE* components in a breeding programme.

## AUTHOR CONTRIBUTIONS

Jaya Basnayake designed the experiment. Sijesh Natarajan conducted the experiments, analysed the data, and prepared the manuscript. Jaya Basnayake, Prakash Lakshmanan and Shu Fukai provided several suggestions and critically reviewed the manuscript. All authors read and approved the manuscript.

## Supporting information


**Figure S1.** Non‐linear relationship between photosynthesis (*A*) versus stomatal conductance (*g*_s_) of all genotypes (A); and transformed *g*_s_ linear relationship with *A* (B).
**Table S1.** Testing equality of slopes assumption for analysis of covariance (ANCOVA) (A); and minimum adequate ANCOVA of *A* with ln(*g*_s_) as covariate (B).

## Data Availability

The data that support the findings of this study are available from the corresponding author upon reasonable request.
