# Supplementary material for: Genotypic variation in intrinsic transpiration efficiency correlates with sugarcane yield under rainfed and irrigated field conditions
Source: Physiol Plant. 2020 Oct 28;172(2):976–89. doi: 10.1111/ppl.13221 (PMC8247395; doi:10.1111/ppl.13221)
Supplement: Supplementary file 1 — Figure S1. Non‐linear relationship between photosynthesis (A) versus stomatal conductance (g s) of all genotypes (A); and transformed g s linear relationship with A (B). Table S1. Testing equality of slopes assumption for analysis of covariance (ANCOVA) (A); and minimum adequate ANCOVA of A with ln(g s) as covariate (B). [file PPL-172-976-s001.pdf]

Supplementary Information

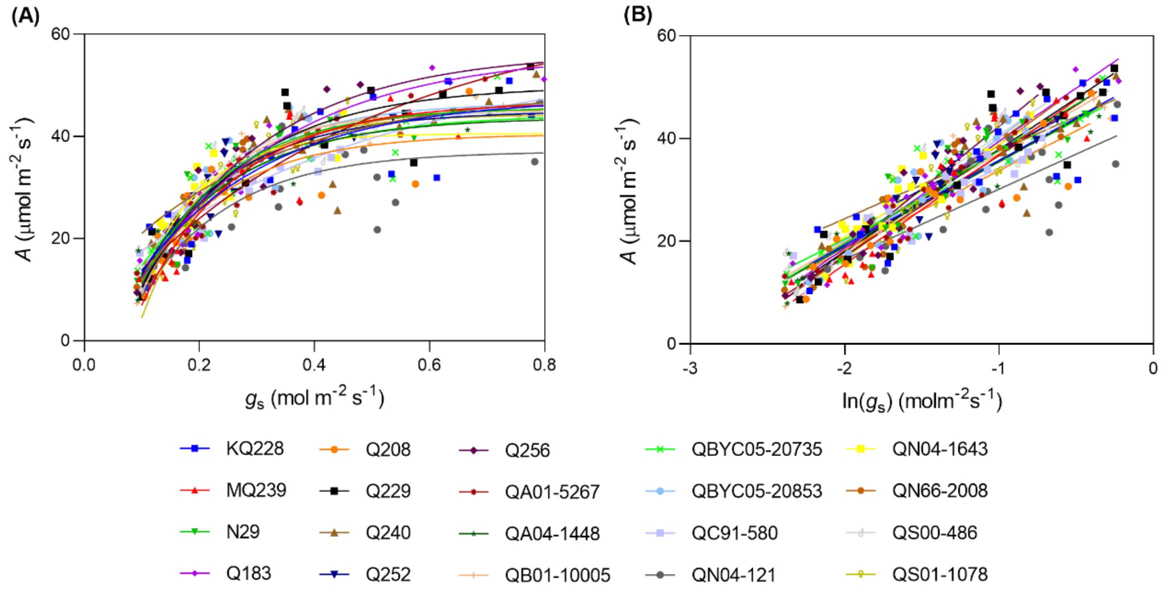

Fig S1: Non-linear relationship between photosynthesis (*A*) vs stomatal conductance (g_s) of all genotypes (A); and transformed g_s linear relationship with *A* (B).

Table S1: Testing equality of slopes assumption for analysis of covariance (ANCOVA) (A); and minimum adequate ANCOVA of *A* with ln(g_s) as covariate (B). ns, not significant; *, *P*<0.05; **, *P*<0.01; ***, *P*<0.001

	***F* Value**	***P***
**(A) Difference in slope**		
Genotype	1.65	*
ln(g_s)	794.66	***
ln(g_s)×Genotype	1.06	ns
**(B) Analysis of covariance for *A***		
Genotype	2.56	***
ln(g_s)	970.39	***
